# Rhabdomyolysis: a manifestation of cyclobenzaprine toxicity

**DOI:** 10.1186/1745-6673-1-16

**Published:** 2006-07-17

**Authors:** Shiven B Chabria

**Affiliations:** 1 Division of Hospital Medicine, Dept. of Internal Medicine, Waterbury Hospital, Waterbury CT, USA

## Abstract

A case of cyclobenzaprine (flexeril) overdose and the resultant rhabdomyolysis is presented. A review of the range of clinical toxicity, management of overdose is described. The similarity of cyclobenzaprine to the tricyclic antidepressant class is emphasized; this report attempts to disseminate related information on this commonly prescribed centrally acting muscle relaxant.

## Background

Cyclobenzaprine (flexeril) after its synthesis in 1961 was found to have limited antidepressant action with no significant advantage over other tricyclic antidepressants[1]. However it was found to act as a centrally acting muscle relaxant and has been widely used ever since. Muscle relaxants account for approximately 18.5% of all prescriptions written for chronic back pain in the United States[2].

We present this case as an example of the range of cyclobenzaprine toxicity and underline the treatment protocols used.

## Case report

A 33 year old male was brought into the emergency room after reportedly ingesting approximately 30 pills of 10 mg each of cyclobenzaprine (flexeril) about 2 hours prior in attempt to commit suicide. Vital signs on admission were 148/88 mm Hg 110/min 37.5 C On initial presentation he was disoriented to time and place and person, the patient was extremely agitated and confused. Airway was well maintained and he did not require intubation. In the emergency room gastric decontamination was achieved with administration of ipecac with resultant emesis but with only 4 pills returning. Thereafter the insertion of orogastric tube was performed and gastric lavage with normal saline was performed till clear return occurred and approximately 7 more pills returned. Thereafter 30 gm of activated charcoal was administered through the tube.

Diagnostic studies were as follows: Hb, 14.6 gm%, Hct, 43.8% WBC 15, 300 with 78% polymorphonuclear leucocytes. Arterial blood gas was as follows PH 7.32 PO2 130 mmHg PCO2 45 mmHg. Chem 10 was unremarkable on presentation, liver function testing revealed elevated ALT of 39 U/L and AST of 30 U/L both normal. The AST peaked to 298 U/L about 12 hours later and trended towards normal over the course of the next few days. Urine toxicity screen was negative, a qualitative screen was positive for cyclobenzaprine a quantitative screen was not available. Creatine Kinase was elevated on admission at 11,963 U/L peaked to 29,840 U/L in 24 hours and trended towards normal in 4 days. EKG showed sinus tachycardia with frequent premature atrial contractions and infrequent PVC's. Chest x-ray and CT scan of the head were within normal limits.

The patient was monitored in the intensive care unit. He was aggressively hydrated, cardiac monitoring showed tachycardia which resolved on day 3 of his admission. The patient remained agitated for 48 hours after presentation and required frequent sedation. Urine output dropped and creatinine rose to 1.8 twenty four hours into his admission, this responded to fluid boluses and creatinine thereafter trended towards normal. His mental status improved gradually to being fully oriented on day 4 and he was transferred to a floor bed. A psychiatry consult opined he wasn't actively suicidal. He was discharged on day 6 and on follow up in the clinic a week thereafter was doing well with no active issues.

## Discussion

Cyclobenzaprine is a muscle relaxant acting primarily on the central nervous system. It is structurally similar to Amitryptilline, differing by only one double bond. Cyclobenzaprine is a weak inhibitor of presynaptic norepinephrine and serotonin. Skeletal muscle relaxant activity is due to brainstem mediated inhibition of gamma motor neurons. Range of toxicity is similar to tricyclic antidepressant overdose. Anticholinergic symptoms predominate, extreme cases may manifest with cardiac dysrhytmias and seizures. Hypo and hypertension have been documented. In a series of 404 cases, adults ingesting less than 100 mg remained asymptomatic. Toxic and Anticholinergic symptoms occurred at doses greater than 100 mg.

Range of toxicity may be manifest by only Anticholinergic symptoms like blurred vision, dry mucous membranes, urinary retention and mydriasis. Tachyarrhythmia's include sinus tachycardia which is very common however even ventricular tachycardia unresponsive to ACLS has been reported. Respiratory failure may develop and in a series of 402 patients about 3% required mechanical ventilation[3]. Delirium, agitation, disorientation and hallucinations have developed even at therapeutic doses. This is especially common in geriatric age group patients[4]. These same symptoms are fairly common after poisoning and overdose[5]. Gastrointestinal effects range from nausea and vomiting to constipation and loss of appetite. High doses might produce hepatic damage with steatosis. Acute renal insufficiency has been reported in case reports[6]. Acid base disturbances manifest as metabolic acidosis. Rhabdomyolysis is an uncommon complication that may develop with prolonged agitation as was most likely in the case presented above. We found only one case description where cyclobenzaprine's range of toxicity was associated with significant Rhabdomyolysis[7]. Psychiatric effects may occur with therapeutic or overdose levels manifesting as agitation, hallucinations, and even precipitation of acute manic psychosis[8].

The management of cyclobenzaprine overdose should follow the same pathway as any tricyclic drug. Gastric decontamination is fairly effective because the Anticholinergic effects of cyclobenzaprine delay gastric emptying and therefore it becomes possible to obtain tablet residues even after significant time elapse. Ventricular arrhythmias QRS widening, or intraventricular conduction abnormalities should be treated with sodium bicarbonate 1 meq/kg IV bolus and repeated if arrhythmias persist this should be followed by IV infusion of sodium bicarbonate to produce an arterial pH of 7.5. The mechanism of action of sodium bicarbonate is unknown. Severe Anticholinergic effects can be reversed with the use of physostigmine salicylate 1 to 3 mg IV. Careful cardiac and hemodynamic monitoring is recommended in the first 48 hours to manage signs of cardiac toxicity and hypotension. Use of physostigmine is not recommended with EKG changes or wide QRS changes[9]
